# Self-care appraisal in nursing assistant students: Adaptation, validation and psychometric properties of the Spanish ASAS

**DOI:** 10.1371/journal.pone.0260827

**Published:** 2021-12-08

**Authors:** Natura Colomer-Pérez, Sergio A. Useche

**Affiliations:** 1 Department of Nursing, Faculty of Nursing and Chiropody, University of Valencia, Valencia, Spain; 2 Faculty of Psychology, University of Valencia, Valencia, Spain; Aalborg University, DENMARK

## Abstract

The core implication of nursing professionals’ labor is promoting self-care and foster well-being among healthcare service users. The beginning of the healing process starts with the provider, and self-care habits are needed to positively impact on patients’ care outcomes at different spheres. Overall, current literature supports the idea that nurses’ personal self-care should be a necessary skill to be expected in their professional role. In this regard, the Appraisal of Self-care Agency Scale (ASAS) is a worldwide known instrument aimed at assessing the ability to engage in self-care. However, it has never been tested in the Spanish context before, and much less in nursing practitioners or apprentices. The aim of this study was to translate, adapt and validate the ASAS for Spanish nursing apprentices, assessing its dimensionality, psychometric properties and convergent validity by means of the Sense of Coherence (SOC-13) questionnaire. **Methods**: Data were collected from a random sample of 921 Certificated Nursing Assistant (CNA) Spanish students and was analyzed trough confirmatory factor analyses via structural equation models. The core ASAS construct and its subscales were correlated with the SOC-13 scores. **Results**: Fair psychometric properties for the questionnaire were set. Also, SEM models endorse the validity and reliability of the four-factor dimensionality of the Spanish adaptation of the ASAS, whose associations to SOC scores were coherent and significant. **Conclusion**: This study allowed to establish that the Spanish version of the ASAS might be a useful tool for addressing self-care-related issues among nursing apprentices, a key population for promoting both their own and patients’ health and welfare through healthy and care-related behaviors.

## Introduction

Educative programs in nursing curricula -university and vocational and educational training- implement formative contents to teach a properly and quality healthcare for others, but what about the attention of those to the care of oneself? The capability to fulfill one’s own self-care requirements was denominated ‘self-care agency’ [1]. Nursing care is provided for patients with health derived or health-related self-care deficits [2] and the construct of ‘self-care agency’ was defined as the human capability to engage in self-care, conditioned by key psychosocial factors such as age, developmental state, life experience, sociocultural orientation, health, and available resources [3]. This construct conforms the central concept in Orem’s Self-Care Deficit Nursing Theory (SCDNT), which highlights on the importance of patient responsibility for self-care behaviors and aims at evaluating patient awareness of health needs, but also seeks to promote a holistic view of self-care.

The so-called *self-care agency* has two fundamental components [4] empowering individuals to get engaged in two stipulated types of action, *i*.*e*., self-care estimative and productive operations. Thus, and with the purpose of measuring this construct through a reliable self-report measurement methodology, the *Appraisal of Self-care Agency Scale* (ASAS) was created. It is based on both power components (the set of specific personal abilities to perform self-care) and operational traits (the ability to organize personal and environmental resources that might be significant in self-care; [1]), and all in all, it is mediated by the idea that performing self-care involves a decision, a choice.

### Self-care agency as a Health Asset

The self-care capacity of each person is developed throughout life, in the course of day to day and through implicit and explicit learning processes. Previous researches that have studied the relationships between ‘self-care agency’ and self-care actions in young students, concluded in their results that there is a strongly significant relationship of prediction of the first concept over the second one [5]. In this sense, the implementation of self-care requires an intentional and calculated action, which is conditioned by the knowledge and repertoire of skills of the person and is based on the premise that individuals know when they need help and, subsequently, they are aware of the specific actions they need to perform and the resources they need to mobilize. However, people could choose non-action, that is, they can decide not to initiate a self-care behavior when it is necessary, for reasons that may include anxiety, fear, or taking other priorities into account. Furthermore, several studies have also found significant associations between ‘self-care agency’, health promotion behaviors and well-being [6, 7] and it was also observed deficit of ‘self-care agency’ associated with risky consumption of drugs and the social and personal consequences deriving from this abuse among European healthcare science students [8].

Accordingly, Vinje [9] advocates for facilitating the student to develop the capacity (the agency) to manage *herself* in a salutogenic way, which is, to identify and mobilize internal and external resources to maintain and improve one’s health, and this is a form of practicing self-care conceiving itself as a Health Asset. Certainly, all those phenomena lead us to understand that the assessment of perceived health self-care should be treated from a comprehensive perspective, considering also the psychological, cultural and social aspects.

Nevertheless, the most prominent challenge implied in this salutogenic perspective is the development of a professional care model oriented towards self-care, patient empowerment, and the enhancement of the professional-based (curative, preventive, protective and promotive) practice. When using this framework, health professionals become change agents and a role model for the population they serve [10–12].

### Self-care agency in nursing science

Professional nursing has the fundamental purpose of promoting self-care and fostering well-being among their patients and health service’s users, as it is related to increasing nurses’ effectiveness and also influence positive patient care outcome. In addition, delivering own self-care activities will increase nurses’ resiliency and may directly impact clinical care [13, 14]. Because professional care-giving relationship serve as role model for the patients and families involved in those operations. Based on these findings, personal self-care should be an expectation of the professional role. Indeed, unhealthy nurses have more absences and are less likely to be able to work at full capacity, potentially increasing the workload for other nurses on the workplace [15, 16]. Thus, encouraging self-care among healthcare professionals may not only benefit the individual healthcare, but it may have an impact on the entire health service. In this sense, whether healthcare professionals must educate people, groups and communities in self-care measures to better manage their chronic diseases or promoting their health status, it is logical to think that they must learn to take care of themselves in order to coherently convey the ability to self-manage one’s care [17, 18]. Therefore, the literature review points towards to enhance current understanding of ‘self-care agency’ operating in nursing professionals (and future ones) and examine strategies to improve their own self-care. The fact of study the self-care appraisal of the future CNAs is paramount in order to provide the possibility of networking, of gathering and organizing knowledge and experience, and to advance the nursing science through the development and validation of instruments aimed at assessing self-care-related factors among them.

In view of the aforementioned consideration, the aim of this study was to translate, adapt and validate the ASAS for Spanish nursing apprentices, assessing its dimensionality, psychometric properties and convergent validity by means of the Sense of Coherence (SOC-13) questionnaire.

## Methods

### Design and study setting

For this study, the goal was to achieve equivalence for Spanish ASAS with a cross-cultural translation. For this purpose, this research project used a traditional forward -and back- translation process, as described by Brislin [19]. Although a first version of original ASAS questionnaire was adapted to South American Spanish (that substantially may differ from the European one [20, 21]), the ASAS has never been adapted in Spain before. The decision to perform this new translation process to Spanish language, -in spite of there are two former cross-cultural validations to Spanish language: the Mexican and Colombian ones- was strictly determined because of the semantic discrepancies found with these both instruments in order to be comprehensible by our Spanish spoken population from Spain.

Therefore, a double translation procedure of the English version of the ASAS was developed through a translation and re-translation (backwards) process, emphasizing on the conceptual -rather than on the linguistic/literal- equivalences. In a first step, two bilingual translators from different regions of Spain -both familiar with the scale terminology-, translated the items of the original English version of the ASAS into Spanish; afterwards, two other bilingual different professional translators translated the new Spanish version of the ASAS back to English (back translation method). The method used for the questionnaire semantic adaptation to the language context of the sample, relied upon a prior pilot study of 42 CNA students using ASAS [22].

Initially, and prior to perform the study, the preliminary translation of item 8 generated some interpretive doubts, easing potential misunderstandings or missing data. Consequently, this aspect was phenomenologically addressed by the main researcher in a focus CNA’s group of expert nurses’ professors in Health Vocational and Educational Training, and the item was finally polished and contextualized, with a high expert-based consensus. Spanish ASAS was retested once more among the same “pilot” group of CNA students, in order to check their understanding of the questionnaire contents.

Finally, since no discrepancies were found, the authors of this study verified the content and semantic equivalence of the translated Spanish version of ASAS scale to the original English version of the scale, and the final version of Spanish ASAS was established.

### Participants

For this cross-sectional research, it was used a full sample of *n* = 921 CNA students voluntarily responding to the questionnaire. The main demographic features of the study sample are presented in the Table 1. For the data collection time, the study partakers were enrolled onto the last semester of the theoretical and practice training program of Nursing Assistant Certificate, and were gathered from the total of public upper secondary schools providing Vocational Education and Training (VET) certifications at the Valencian Community (Spain). A total of 23 public schools offers this certification, and the educational system, the curricula and the training program are the same for all educative centers. The whole study population (including all these schools) comprises a total of *n* = 1150 individuals.

**Table 1:** Sociodemographic characteristics of the study participants.

Variable	Group/value	n	%
**Gender**	Male	150	18.46
	Female	771	81.54
**Geographical context**	Rural areas	66	7.17
	Urban areas	520	56.46
	Large cities	335	36.37
**Family income-level**	Low	283	30.68
	Medium/Low	261	28.36
	Medium	297	32.19
	Medium/High	64	6.99
	High	16	1.78
**Current employment situation**	Employed	222	24.11
	Unemployed	699	75.89
**Career choice’ main motivation**	Vocational motivation	444	48.21
	Impossibility of access to other studies	21	2.28
	Seeking better work	316	34.31
	No motivation	25	2.71

An a priori statistical power analysis allowed to establish a minimum sample size of *n* = 820 subjects assuming an effect size (*f*) of 0.098 as per de pilot study analyses, an alpha (*α*) level = .05 and a power (*ß*) = 0.80 [23]. As it covered almost the whole study population, a non-probabilistic method was used, as the research team directly contacted with all the potential institutional partakers, *i*.*e*., educational centers offering this program across the region, all of them accepting to partake in the study. At the individual level, 80% of CNA students accepted to collaborate and filled up the questionnaire form.

The sociodemographic data gathered from participants consisted of: *a*) gender (male, female), *b*) age (*M* = 28.52; *SD* = 11–42 years), *c*) current occupational status, *d*) income level (understood as the net level of income received), *e*) geographical emplacement of their educational centers (*i*.*e*. rural, urban, large city), and *f*) motivation of study choice (*i*.*e*. vocational, could not be enrolled in other program, seeking for better employment options, or just unmotivated). More sociodemographic information about the sample (study participants) is available in Table 1.

### Appraisal of self-care agency scale, ASAS

The ASAS scale was initially developed as a shared effort of the department of nursing sciences of the University of Limburg, the Netherlands, and of Wayne State University, Detroit, Michigan, USA [24]. The project arose in 1984 and the questionnaire was considered to meet requirements to be tested in August 1985. The aim was to measure the core construct of Orem’s self-care theory in nursing, assessing whether a person -adults over 18 years old suffering from different health situations- actually could meet (or not) their general self-care needs [2, 25]. At the present time, there are several different scales available to assess either the self-care ability or the self-care agency (both terms are used to operationalize the ‘self-care agency’ construct) over adults in the world [26]. It is presented in two versions (24-item and 15-item); notwithstanding, the most widely used measure is the 24-item version of ASAS. The scale has been translated and adapted-validated to several languages by means of studies conducted in countries such as Hong Kong [27], Norway [25], United States [28], etc. The reliability assessed got Cronbach’s alpha values between [α = .70 - .90] in those previous several studies in which it was applied. Regarding the first translation of the ASAS into Spanish language, it was carried out by professional experts in Mexico [20], and a second cross-cultural validation was carried out with Colombian patients [21], which concluded to support the use of the ASAS in Spanish speakers from Colombia as a consistent and reliable instrument. The scale is comprised by 24-items on a Likert scale with five alternatives, between being ‘strongly disagree’ that coincides with the lowest value of agency, and ‘strongly agree’ being the highest. Each individual can get a real score range from 24 to 120 points. Greater scores indicate a better capability to take care of personal health and procure well-being for oneself. Additionally, there are three items questioned in a negative way which must be recoded before the data analysis.

### Data processing

Preliminary power analysis calculations were carried out with G*Power (version 3.1.9.6 [23]). Once the data was collected, listwise deletion was used to filter incomplete or non-filled questionnaires, as very few questionnaires (<10 forms) had any missing data, while the sample size remained considerably large, for a total of 921 fully filled questionnaires. Subsequently, basic analytic procedures on the sample features were carried out.

After an initial descriptive analysis of the data, ASAS scores were obtained, and Brown-Forshyte robust mean tests were performed, in order to determine potential gender-based differences in its dimensions. The performance of such robust analyses is suggested when: *(i)* Fisher’s F test for ANOVA is not suggestable due to the absence of normality and/or homoscedasticity, and *(ii)* sub-sample sizes are quite unequal or disproportional due to characteristics of the sample (we counted on a greater number of female participants). Further, the factorial structure of the ASAS was tested through a rigorous process, through competitive Confirmatory Factor Analyses (CFA) with successive fit steps (forward), testing different factor solutions of the questionnaire.

Given than many theoretical and empirical approaches to the ASAS were already available confirming the hypothesized structure or factor composition [27, 29–33], this study used confirmatory models. CFA also involves many strengths for what concerns the treatment of missing cases (*i*.*e*., omitted responses), categorical variables and non-normally distributed data [34]. Moreover, one critical benefit of competitive confirmatory factor analyses is the capability of testing several models under different theoretical suppositions and hypothesized configurations, denoting what solution has a more suitable and scientifically parsimonious fit. In this case, the statistical package IBM SPSS AMOS (version 26.0) was employed for specifying and estimating the SEM models used for data analysis.

The cut-off criteria for considering an item’s factor loading as adequate was *λ*> 0.30. As advised in advanced sources, the model fit was weighed through several estimators and indexes from different logics and families, based on the cut-off standards proposed by Marsh as “rules of thumb” for this matter [35]. Particularly, most of the available statistical indices suggested for this estimation method were used, as described below:

*Root Mean Square Error of Approximation (RMSEA)*, a badness-of-fit measure helping to assess how far a theoretically plausible (hypothesized) model could be from an *ideal* model [36].

*Confirmatory Fit Index (CFI)*, developed by Bentler, it consists of a normed index compared the baselined model with the hypothesized one [37].

*Tucker-Lewis Index (TLI)*, that assesses a relative decrease in misfit per degree of freedom contained in the model [36].

*Normed Fit Index (NFI)*. Evaluates model fit by means of comparing a model of in reference to a model of completely uncorrelated variables [38].

As for the interpretation guidelines for these indexes, it is commonly accepted that a CFI/NFI/TLIs greater than .90 and RMSEAs lower than .08 (although better if < .05) suggest (although does not warranty) a satisfactory model goodness-of-fit. Therefore, the models’ suitability was also tested in consideration of the strength and coherence of the obtained estimates, enhanced by the absence of large or redundant modification indexes.

The internal consistency (or reliability) of the questionnaire and its items was assessed through three different indexes: (*i*) Cronbach’s Alpha (α); (*ii*) McDonald’s omega (ω), having the advantage of taking into account the strength of association between items and factors and item-specific measurement errors, providing more realistic estimates on scale reliability [39]; and (*iii*) Composite Reliability Index (CRI) an additional coefficient ranging from 0 (zero consistency) to 1 (full consistency), mathematically based on the factor loadings and residuals seen in the results of SEM-based confirmatory analyses (CFAs) [40]. As additional reliability measures, both ω and CFI also contribute to overcome the conventional shortcomings of Cronbach’s α_s_ if used as a single indicator to test scale reliability, greatly dependent on fixed loadings for its calculation [40–43].

As the assumption of multivariate normality could not be met with the present data, that was preliminary ordinal, and it can to (*e*.*g*.) lead to inflate *X*^2^ (Chi-square) values, and/or to underestimate standard errors, enhancing potentially incorrect inferences at the moment of testing model parameters [44], the model was bootstrapped through a Monte Carlo (parametric) procedure. Bootstrap estimation is a re-sampling technique by which multiple subsamples of an identical size are randomly used to test a model, favoring that (*e*.*g*.) the results of the estimates may be bias-corrected, do not present problems of normality, and type I errors (false positives) in regression paths can be avoided, and constitutes a reasonable alternative to other estimation methods such as Satorra-Bentler or Weighted Least Square Mean and Variance adjusted (WLSMV), that cannot be performed with AMOS software.

Finally, the convergent validity (coherence of the relationship between the studied constructs and theoretically associated variables) of the ASAS was assessed by means of a Criterion Variable (CV) supported by the literature to be related with self-care habits, *i*.*e*., sense of coherence, measured through the SOC questionnaire [45], that was correlated to the ASAS dimensional scores through Pearson’ bivariate correlations, in order to test the directional coherence and significance of the associations among them.

### Ethics statement

In order to perform this research, all permissions needed were granted from both the educational centers and the competent organism in the area of education government in the region. The Autonomous Secretariat of Education and Research of the *Conselleria d’Educació*, *Investigació*, *Cultura i Esport* (institutional review board) certified that the study was designed in accordance with the current Spanish and European data protection protocols and ethical guidelines for research involving human subjects (IRB number: 05ED01Z/2016/406/S).

Likewise, the paper questionnaires were exclusively identified by means of a generic code, in order to guarantee the confidentiality and anonymity of the information given by participants. Before answering the questionnaire, all center staff members and students have read and agreed in writing with the informed consent. This consent included the relevant information required by the IRB, *e*.*g*., the purpose of the research and clarification on the strictly scientific use of the data to be collected.

## Results

### Descriptive outcomes of ASAS

The average value in the ASAS scale was *M* = 92.09 (*SD* = 10.53), ranging the scores between [*M* = 44–120]. As for the ASAS full-score, females (*M* = 92.64; *SD* = 10.35) reported significantly higher scores than males (*M* = 89.66; *SD* = 11.04), with *F*_(1,240.73)_ = 10.37; *p*< .001. Also, female nursing students have shown greater scores than males for two subscales of the ASAS: “Health Behavior” (*F*_(1,240.73)_ = 10.37; *p*< .001) and “Health Awareness” (*F*_(1,264.19)_ = 3.96; *p*< .05), while male students reported significantly higher scores for the factor “Ignorance” (*F*_(1,234.64)_ = 13.78; *p*< .001).

No sex-based significant differences were found for the case of the factor “Resources”. The full set of descriptive values reported in the ASAS and its dimensions is available in Table 2.

**Table 2:** Descriptive values and gender-based robust mean comparisons of the ASAS and its main components.

Variable	Group	N	Mean	SD^a^	SE^b^	95% CI^b^		Range		Robust Mean Comparisons (Brown-Forshyte)			
						Lower	Upper	Min	Max	Statistic^d^	df1	df2	Sig^e^
**ASAS (Total)**	**Total**	921	92.09	10.53	.35	91.41	92.77	44.00	120.00	10.37	1	240.73	< .001
	Female	751	92.64	10.35	.38	91.90	93.38	44.00	120.00				
	Male	170	89.66	11.04	.85	87.99	91.33	57.00	118.00				
**Health Behavior**	**Total**	921	4.26	.54	.02	4.23	4.30	1.50	5.00	15.69	1	234.89	< .001
	Female	751	4.30	.53	.02	4.26	4.34	1.50	5.00				
	Male	170	4.11	.59	.04	4.02	4.20	2.20	5.00				
**Ignorance**	**Total**	921	2.75	.51	.02	2.72	2.79	1.00	4.30	13.78	1	238.64	< .001
	Female	751	2.72	.50	.02	2.69	2.76	1.00	4.30				
	Male	170	2.89	.54	.04	2.81	2.97	1.00	4.20				
**Health Awareness**	**Total**	921	3.09	.71	.02	3.05	3.14	.00	4.00	3.96	1	264.19	< .050
	Female	751	3.11	.71	.03	3.06	3.16	.00	4.00				
	Male	170	3.00	.67	.05	2.90	3.10	1.00	4.00				
**Resources**	**Total**	170	3.78	.66	.05	3.68	3.88	2.30	5.00	1.33	1	248.39	.251
	Female	751	3.85	.65	.02	3.80	3.90	1.70	5.00				
	Male	170	3.78	.66	.05	3.68	3.88	2.30	5.00				

Notes for the table

^a^ Standard Deviation

^b^ Standard Error

^c^ Confidence Interval with lower and upper thresholds at the confidence level of 95%

^d^ F-test value

^e^ *p*-value of the test.

### Structural equation modeling outcomes

With the purpose of better-understanding the factorial dimensionality of the Spanish version of the Appraisal of Self-care Agency Scale (ASAS) in consideration of various possible structures, four basic competitive theoretical-based CFAs were carried out. Firstly, an unifactorial structure working under the assumption of a single Self-care Agency-based factor was assessed. Secondly, and as self-care agency encompasses two fundamental components (*i*.*e*., empowering and productive operations) that may eventually serve as high-order dimensions [4], a bifactorial composition. Of the ASAS was also hypothesized. Thirdly, it was examined the original structure composed of a four-factor structure, in order to develop fit comparisons and thus determine the best possible and most parsimonious factor structure for the scale. Finally, a five-factor model including the complementary factor *ability* was tested, in accordance to the reported by Söderhamn & Cliffordson [31], in which this fifth factor is calculated on the basis of four items belonging to other subscales of the questionnaire.

The model fit for the first solution tested (one single factor) was considerably inconsistent and poorly fitted, while the baseline four-dimensions model showed better fit indexes (see Table 3). A careful examination of this unconstrained four-dimensional model allowed us to recognize a short set of very large modification indexes suggesting a relevant relationship between some few questions, plus items presenting obvious psychometric glitches (λ< 0.3). The new simplified four-factor constrained model fitted the data considerably well, containing adequate goodness-of-fit indices, as presented in Table 3.

**Table 3:** Competitive CFA–goodness-of-fit indices obtained for the structural models.

Data	Model	*X* ^2^	*p* ^a^	RMSEA^b^	90% CI for RMSEA^c^		CFI^d^	NFI^e^	TLI^f^
					Lower	Upper			
Full sample (*n* = 921)	1. Unifactorial solution	1662.237	< .001	.078	.075	.082	.727	.695	.701
	2. Bifactorial solution	1447.489	< .001	.080	.077	.084	.745	.715	.717
	3. Four-factor baseline model	1502.354	< .001	.075	.071	.079	.757	.724	.724
	4. Four-factor adjusted model (retained)*	431.397	< .001	.043	.038	.048	.944	.915	.919
	5. Five-factor model (including the factor *ability*)	2419.459	< .001	.044	.039	.049	.945	.917	.915
First half (*n* = 460)	1. Unifactorial solution	963.190	< .001	.089	.083	.094	.709	.658	.678
	2. Bifactorial solution	943.137	< .001	.088	.082	.093	.716	.665	.685
	3. Four-factor baseline model	902–247	< .001	.078	.073	.084	.775	.721	.744
	4. Four-factor adjusted model (retained)*	341.663	< .001	.050	.043	.057	.929	.911	.906
	5. Five-factor model (including the factor *ability*)	416.965	< .001	.044	.039	.049	.945	.918	.916
Second half (*n* = 461)	Four-factor adjusted model (retained)*	328.362	< .001	.048	.041	.056	.934	.902	.904

Notes for the table

^a^ All *p*-values were lower than .001

^b^ Root Mean Square Error of Approximation

^c^ Confirmatory Fit Index

^d^ Confidence Interval

^e^ Normed Fit Index

^f^ Tucker-Lewis Index.

It is worth highlighting that, if this model fit is compared with the unifactorial and bifactorial solutions with the same set of items, the final four-factor structure has a much better fit, even without the need of removing an extensive set of questionnaire items: only two questions (*i*.*e*., ASAS7 and ASAS12) presenting considerably low factor loadings (*λ*< 0.30) and obvious psychometric problems were removed, and the scale. Therefore, this new 22-items composition had an optimal fit and structure, keeping in mind both (*i*) the overall acceptable and adequate factor loadings (all *λ*> 0.35) and (*ii*) the good consistency and reliability scores obtained in further analyses for all the four factors composing the retained model (as shown in Table 4).

**Table 4:** Item descriptive—factorial composition and bootstrapped bias-corrected indices of the retained four-factor model for the ASAS.

Item	Nature	Factor	M^a^	SD^b^	λ^c^	S.E.^d^	C.R.^e^	*p* ^f^	Bootstrap bias-corrected values^g^				
									Estimate^h^	S.E.^d^	95% CI^i^		*p* ^j^
ASAS1	+	Factor 1: Health behavior (6 items)	4.010	.923	.644	.081	13.231	< .001	1.069	.081	.941	1.217	< .010
ASAS3	+		4.180	1.023	.361	.064	9.690	< .001	.623	.064	.523	.741	< .010
ASAS4	+		4.430	.813	.561	.062	12.383	< .001	.769	.066	.664	.886	< .010
ASAS5	+		3.930	.952	.669	.066	16.114	< .001	1.071	.068	.968	1.195	< .010
ASAS8	+	CRI^k^ = .963	4.790	.649	.351	.041	9.435	< .001	.385	.044	.313	.457	< .010
ASAS21	+	α^l^ = .727 ω^m^ = .690	4.250	.909	.611	.071	13.231	< .001	.936	.074	.822	1.063	< .010
ASAS2	-	Factor 2: Ignorance (6 items)	2.345	1.018	.729	.068	10 .639	< .001	.728	.068	.603	.911	< .010
ASAS6	-		3.623	1.326	.421	.105	7.249	< .001	.760	.115	.585	.952	< .010
ASAS11	-		2.688	1.354	.366	.103	6.505	< .001	.672	.110	.500	.864	< .010
ASAS13	-		2.615	1.249	.411	.098	7.122	< .001	.701	.157	.466	.982	< .010
ASAS20	-	CRI = .944	2.999	1.273	.380	.102	6.400	< .001	.654	.115	.505	.895	< .010
ASAS23	-	α = .671 ω^m^ = .690	2.245	1.128	.898	.129	10 .639	< .001	1.374	.172	1.098	1.658	< .010
ASAS14	-	Factor 3: Health Awareness (3 items)	4.368	.909	.734	.074	12.397	< .001	.914	.074	.787	1.057	< .010
ASAS15	-	CRI = .946	4.031	1.030	.576	.073	12.291	< .001	.895	.084	.779	1.060	< .010
ASAS24	-	α = .614 ω^m^ = .613	3.884	.988	.731	.088	12.397	< .001	1.095	.099	.946	1.270	< .010
ASAS9	+	Factor 4: Resources (7 items)	3.450	1.274	.477	.134	9.911	< .001	1.324	.134	1.129	1.590	< .010
ASAS10	+		4.010	.950	.453	.068	10 .475	< .001	.711	.068	.608	.825	< .010
ASAS16	+		3.800	1.124	.545	.079	12.681	< .001	1.004	.081	.875	1.137	< .010
ASAS17	+		3.290	1.380	.426	.097	10 .048	< .001	.970	.092	.840	1.134	< .010
ASAS18	+		4.310	.894	.506	.069	10 .739	< .001	.746	.070	.644	.874	< .010
ASAS19	+	CRI = .953	3.940	.944	.671	.082	12.787	< .001	1.043	.082	.924	1.189	< .010
ASAS22	+	α = .697 ω^m^ = .703	4.040	1.048	.437	.076	9.911	< .001	.755	.080	.629	.886	< .010

Notes for the table

^a^ Mean

^b^ Standard Deviation

^c^ Standardized factor loading

^d^ Standard Error

^e^ Critical Ratio

^f^ All *p*-values were lower than .001

^g^ Bootstrapped (bias-corrected) model

^h^ Unstandardized estimates

^i^ Confidence Interval at the level 95% (lower bound–left; upper bound–right)

^j^ All *p*-values in bootstrap were lower than .010

^k^ Composite Reliability Index

^l^ Cronbach’s alpha

^m^ McDonald’s omega.

Further, when the complementary fifth factor (*ability*) was tested through the adding of a fifth factor built up by means of using four items already loading in other factors, a similar model fit was determined, but the model had qualitatively a lower consistency and parsimony for three key reasons: first, since two out of the four items composing the subscale (*i*.*e*. ASAS7 and ASAS12) had considerably low factor loadings (*λ*< 0.30) and had to be deleted from the factor; secondly, because simultaneously loading two different factors impairs the psychometric value of these items for their original dimensions (*λ*s considerably decreased); and finally, due to the low reliability of the fifth factor calculated, composed of two items whose consistency was substantially low, with an α< .500 and CRI< 700.

Complementarily, and as a manner to corroborate what was found working with the full sample, the dataset was split in two halves. All the models were verified with the first sample half (460 individuals), finding that–same as with the full sample–the best fit corresponded to model 4 (four-factor), remaining as the most parsimonious and psychometrically rigorous, even if compared to model 5 (five-factor model). Also, it is worth considering that the 5-factor model did not add considerable improvements to the scale structure and impaired the psychometric properties of other dimensions, reason by which the four-factorial structure of the ASAS was retained as the best confirmatory model possible for the current data. Afterwards, a second inspection to the data fit to model 4 was performed with the second sample half (461 individuals), finding that the goodness-of-fit indexes were kept with similar values and features.

In regard to the bootstrapping procedure, that was applied to the retained model in order to correct possible impaired outcomes, that could be biased as a consequence of (*e*.*g*.) the lack of multivariate normality of the data, it was found that all the regression paths (*i*.*e*., relationships between each item and the factor it belongs to) remained significant at a *p* < .010 level. Further, all these paths kept their coherence, directionality and factor loading to the hypothesized dimensional composition of the four-factor validated version of the ASAS. Bootstrapped model estimates (*i*.*e*., unstandardized), their standard errors, confidence intervals at the 95% level and p-values are presented in detail in Table 4.

#### Internal consistency of the ASAS

**Fig 1:**
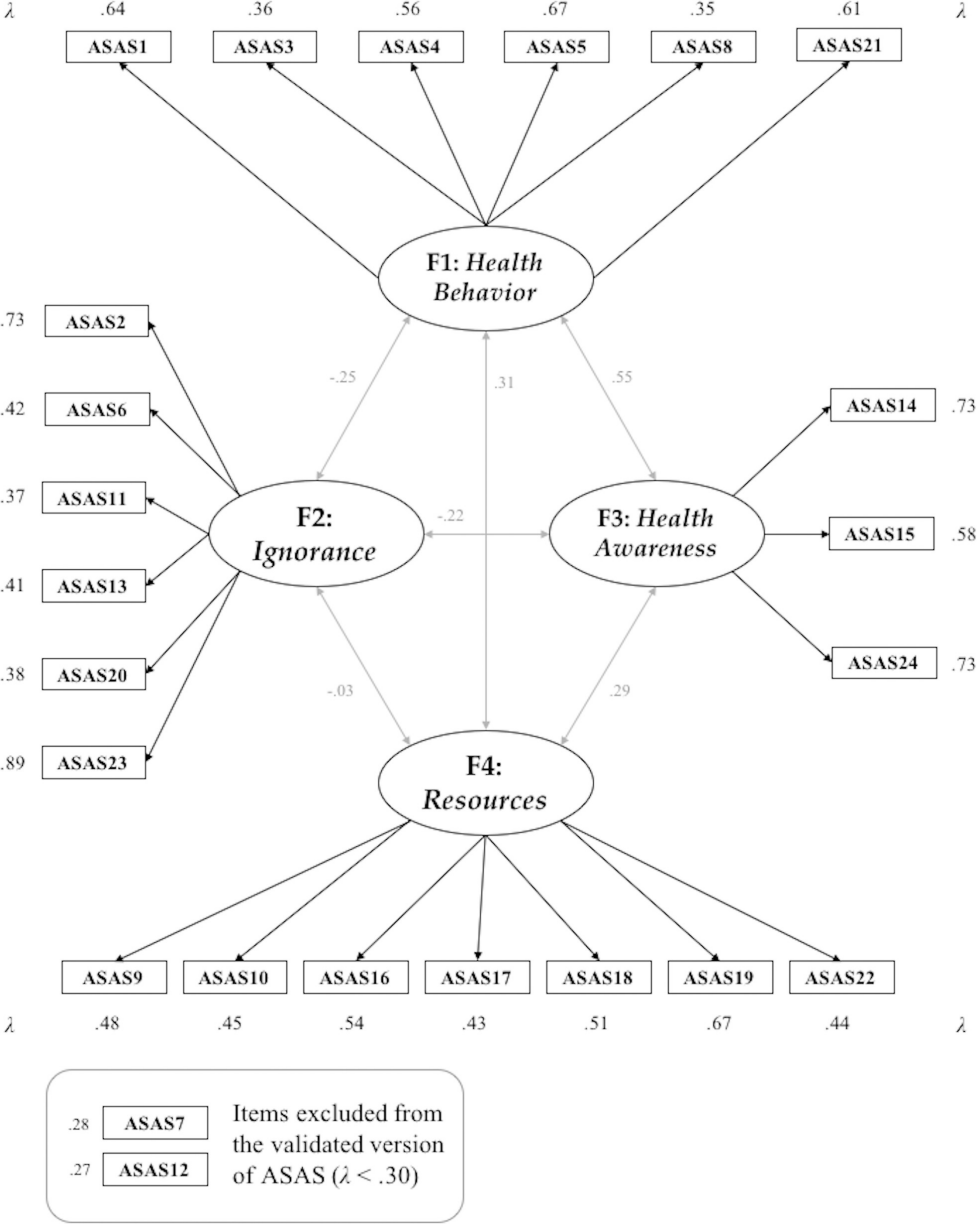
Standardized parameter estimates and factor correlations. *Notes*: All standardized estimates were *p <* .001; the numbers within squares represent the original numbers of the items in the ASAS (as shown in Table 4).

Regarding internal consistency, all the four dimensions of the retained model have shown acceptable-to-optimal values, as follows: (*i*) Cronbach’s alpha coefficients (α) were all acceptable (although not optimal), being all of them above .600, ranging between [.603 - .727]; (*ii*) McDonald’s omega (ω) coefficients were also acceptable (although not optimal), all over .610, oscillating between [.613 - .703]; and (*iii*) Composite Reliability Indexes (CRIs) were all optimal, always between [.944 - .963], for each one of the four factors composing it. as reported in Table 4 and graphically at Fig 1.

Apart from these coefficients, Table 4 also presents in detail the content, descriptive data (arithmetic means and deviations), standardized factor loadings or “lambda” coefficients (*λ*s) and significance levels (all *p* < .001) of each one of the items composing the ASAS, being all factor loading coefficients in the retained model large, positive and significant at their correspondent dimensions.

### Convergent validity: Dimensional correlations with SOC

Finally, the convergent validity analyses, carried out through assessing the bivariate correlations between the Sense of Coherence Questionnaire (SOC-13) and each one of the ASAS factors (*i*.*e*., F1 –Health behavior, F2 –Ignorance, F3 –Health awareness, and F4 –Resources) were performed. Bearing in mind the current evidence, provided by studies simultaneously using both scales, it was expectable to find positive and significant associations between ASA-related scales and the unidimensional SOC-13 score, except for the “Ignorance” subscale (F2), whose calculation has an inverse nature, as also empirically tested in previous research [45–47].

Accordingly, the obtained correlations show significant and coherent associations between each ASAS dimension and the overall score of the SOC (except for F2 –Ignorance, whose correlation is trendy negative, although non-significant), endorsing the convergent validity and practical sense of the questionnaire in its factorial structure, in addition to the reliability and fit indexes previously indicated [45]. Table 5 displays the associations observed between ASAS dimensions and the criterion variable, including Pearson’ *r* scores and significance levels.

**Table 5:** Convergent validity (bivariate correlations) between ASAS dimensions and SOC-13 questionnaire score.

Factor		Statistic	F2	F3	F4	CV
**F1**	Health Behavior	*r*	-.248**	.554**	.664**	.312**
		*Sig*.	*<* .*001*	*<* .*001*	*<* .*001*	*<* .*001*
**F2**	Ignorance	*r*	1	-.223**	-.208**	-.029
		*Sig*.	*--*	*<* .*001*	*<* .*001*	.*384*
**F3**	Health Awareness	*r*		1	.625**	.292**
		*Sig*.		*--*	*<* .*001*	*<* .*001*
**F4**	Resources	*r*			1	.356**
		*Sig*.			*--*	*<* .*001*
**CV**	Sense of Coherence	*r*				1
		*Sig*.				*--*

Notes for the table

** Correlation is significant at the 0.001 level (2-tailed)

* Correlation is significant at the 0.050 level (2-tailed).

## Discussion

The present study sought to analyze the psychometric properties of the ASAS and to test the validity of its Spanish version in a sample of CNA students, population in which ‘self-care agency’ is particularly relevant in order to optimally face future workload stressors. Results of this study provide further evidence that the instrument is a reliable and valid measure for assessing self-care among nursing assistant students and exhibited adequate psychometric characteristics and good-fit-indexes.

The ASAS has been tested, retested, and found to be a transcultural instrument in measuring ‘self-care agency’ among different population, but only the ASAR, the revised version of 15-item [48], had been used to assess self-care in Spanish samples before. Moreover, only a few studies have taken the next steps to examine the factor structure underlying the Swedish [31], the Chinese [27], and the American [49] versions of ASAS. However, no studies of factor analysis of ASAS in Spanish population have been carried out so far. The Four-Factor model including “Health behaviour” (6 items), “Health awareness” (3 items), “Ignorance” (6 items), and “Resources” (9 items) fitted well to the data and remained statistically adequate, which suggests that ASAS constitutes a solidly-structured instrument, that is also highly suitable for (in this case) assessing the reported self-care levels among CNA students. If comparing the results obtained in this study with other factor analysis of 24-item ASA, we found differences and similitudes with them. Those obtained by Söderhamn [31], the first original factor analysis of ASAS study, considered to influence four latent variables which reflected an initial 4-dimension of ASA Finnish version scale; “Health Behaviour” factor could be also used as a separate scale, since its Cronbach’s alpha reliability coefficient was high enough to allow for comparisons on the individual level.

In a deeper analysis step of the Swedish study, it was found some items reflecting a fifth new latent variable they labelled the “Ability” factor, a critical component which was finally included in the model. The second study assessing the factor analysis was conducted in Hong Kong and included a Chinese version of the ASAS [27]. It was modified to include 2 new items on the prior one to conducting translation and the factor analysis; therefore, a 7-factor structure that accounted for 68% of the total variance and internal reliability of .72 was built. Taking into account the third American factor analysis, the item analysis of the original ASAS in English had not yet been reported and a 2-factor dimensions of the scale was reported in a sample of 141 diabetes-mellitus patients. This factor structure solution accounted for 39.98% of the covariance among a new 20-item scale with a reliability of .85.

### Psychometric highlights on the 4-factor dimensionality of ASAS

The present study revealed that 4 factors were important to maintaining the care for oneself among certificate nursing assistant students, including also the acquisition and performance of different activities that can be considered as necessary to achieve and promote well-being. In regard to other recent studies performing path analysis of ‘self-care agency’, we find the Turkish validity of the scale [50], and the study of Tanimura [51] identified a causal relationship between ASAS and healthy behavior among community-dwelling elder people in Japan and confirmed the potential to adopt positive and proactive healthier behaviors if people acquire ‘self-care agency’. Waki [52] used an adaptation of ASAS and found that a ‘body self-awareness’ served as an intermediary factor that enabled the performance of self-care operations by making the most use of self-care agency. Many other studies using ASAS in clinical context had been implemented [53–62].

As regards evidence of convergent validity, the dimensions that make up the ASAS in our study (health behaviour, health awareness, ignorance and resources) were significantly correlated with sense of coherence (SOC). A significant positive correlation was shown between sense of coherence and ASAS positive factors (F1, F3 and F4), and a negative correlation between sense of coherence and the negative ASAS factor (F2). When it comes to comparing the connections between both constructs, few studies were found, but the first findings by Sonninen [63] confirmed significant correlations between Finnish ASA scale and SOC among home dwelling people. Since that, Söderhamn [64] confirmed that patients with high risk for undernutrition had low self-care ability and weak SOC; at a later stage, showed that elder rural people with a strong SOC were quite able to further develop features that influence self-care and health in their own contexts in a phenomenological study [65]. In other clinical contexts, a study showed that patients dialyzed at home scored higher (*i*.*e*., better) on both, ASAS and SOC, than those dialyzed in center [66]. Fex [45] also confirmed a positive linear relationship between ASAS and SOC among people using advanced medical technology at home, showing that high SOC is a positive contributing to optimal ‘self-care agency’. Other recent research [67] has also related both constructs, finding a synergy between the improve of ‘self-care agency’ and sense of coherence in a better manage of systemic lupus erythematosus in young patients in Indonesia.

### Implications for the nursing practice

According to nursing practice implications, the knowledge that CNA students showed strong levels of ‘self-care agency’ during their apprenticeship could be translated in interpreting, understanding, and promoting self-care throughout the CNA healthcare career. The benefits of mastery of self-care include responsibility, control, independence, autonomy, copying skills, increase healthcare knowledge, well-being, and improve quality of life [26, 68–71]. Previous findings exploring ‘self-care agency’ among people suffering from different pathologies are in accordance with the idea that this dimension of self-care must be tackled by nursing professionals in holistic contexts of health promotion [72]. Göransson [73], monitoring data in specific app managed by nurses, concluding that this support gave more security and increased the self-care ability among users. Finally, Wong [61] also addressed for nursing educational interventions developed both, in primary and secondary school in order to improve the student’s self-care agency. In this sense, a research on specific patients [74] concludes that nurses have the responsibility to engage in self-care patients process taking into account that neglecting the importance of ‘self-care agency’ as a basic element of caring process, disfavor the ability of self-care in patients. Because nurses must be encouraged not only to display self-care abilities, but to design educational and training programs that include care services so that patients can acquire independence in activities of daily living in order to improve their ‘self-care agency’.

All in all, nurses have a crucial role in managing the counselling to affect patients’ self-care agency in a positive direction along the clinical practice. Nurse educators teach nursing students to take care of patients in community and inpatient settings. However, minimal emphasis is given to teaching nursing students how to provide self-care to themselves [75]. For this purpose, professors both in vocational and educational training (nursing assistant certifications) and at universities (faculties of nursing) must function in a supportive-educative role promoting self-care among their students. According to this study point of view, measuring ASAS must be a good approach in order to search for a diagnose of a lack of self-care among these nursing students, as the first step to address self-care to themselves, and furthermore, to be capable to provide strategies to self-care in future healing environments.

### Conclusion

This study supports the assumption that ‘self-care agency’ constitutes an operational concept that encompasses key issues, such as cognition, attitude, knowledge, and motivation to be translated into the practice. These variables can directly affect the care-related work of both nurses and CNA professionals, but also can be increased by nursing professors’ support and specific learning approaches.

This study showed how the validated version of the ASAS represents a suitable self-report instrument to assess ‘self-care agency’ among CNA students, with an optimized structure fitted to four dimensions, and a large set of satisfactory indicators of validity and test reliability.

One way to address the well-being of nurses is to incorporate self-care interventions early in nursing curricula and maintain those interventions throughout the program in order to better understand and value personal self-care. In this sense, promoting ‘self-care agency’ among nursing assistant certifications in early stages of apprenticeship has been shown to positively enhance an individual’s health-promoting behaviours and moreover, it is related to the development of a strong sense of coherence, that is subsequently reinforcing the better understanding and manageability of capabilities for self-care.

### Limitations of the study and further research

Although the study sample was considerably large and all the basic statistical parameters were successfully met during the analysis phase, there are some potential limitations that should be acknowledged in the case of this research. Firstly, one key limitation is related to the cross-sectional study design, which hampers the ability to make causal inferences. Even when that questionnaire-based designs help in terms of practicality and accessibility to large samples of respondents, they may enhance several types of bias, including common method variance and acquiescent responses, that are rather typical from self-reported information on topics that may result sensitive for participants. As key methodological remedies, we (*e*.*g*.) ensured the anonymity of the study, also using a questionnaire encompassing both positive and negative items, that also has shown good reliability indexes. However, we still count on a single measure, and test retest reliability of the translated version of the ASAS remain pending to be assessed.

Secondly, longitudinal studies should be conducted to confirm the stability of this scale. Also, it would be of interest to continue testing the proposed model and expand the study of its psychometric properties to samples with different nurse’s features and professional profiles (CNA and RN) and in active employment nurses in order to get adaptations adjusted to these contexts. Another key point to consider is the gender-based disproportionality of the sample, fact which prevent us to perform further comparisons and invariance-related analyses on this validated version of the ASAS. Therefore, it becomes relevant to encourage other researchers to retrieve enough data to explore this issue, that (given that nursing-related occupations are still highly gendered) remains almost undisclosed in the current times. Finally, it is worth remarking that there is a lack of studies not only among CNA and nursing professionals or trainees, but also among other groups of healthcare providers; thus, further empirical investigation is required focusing on ‘self-care agency’ -evaluated with ASAS- related to healthcare professionals undergoing training.

## Supporting information

S1 Dataset Raw data is available in the file (database) attached to the electronic version of this manuscript. (SAV)
